# Phage Display Libraries Identify T Cells

**DOI:** 10.1371/journal.pbio.0020117

**Published:** 2004-04-13

Doctors and researchers often look for the rapid proliferation of T cell populations, key defensive players in the immune system, as a telltale sign that the body is working hard to fend off a foreign threat. Every one of these circulating white blood cells carries a T cell receptor (TCR) that binds to a specific protein, or antigen, when displayed on the surface of a cell. A match between TCR and displayed antigen results in the cell's death and the subsequent expansion of T cell clones, all programmed to recognize the original offending protein. Some TCRs bind and expand in response to pathogenic antigens, such as viral or bacterial proteins. But T cells can also react and proliferate inappropriately in response to the body's own proteins, leading to destructive autoimmune diseases such as multiple sclerosis, which is characterized by immune system attacks on nervous tissue. Self-recognizing TCRs, however, can also target and destroy tumors—though full activation of these T cells is inconsistent and poorly understood.[Fig pbio-0020117-g001]

**Figure pbio-0020117-g001:**
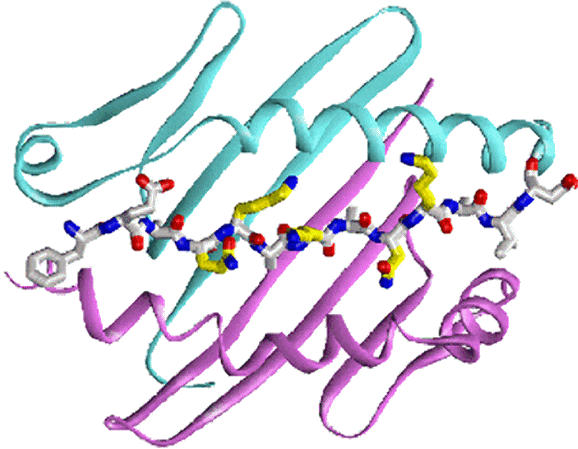
Peptide display

Identifying the particular antigen behind an exploding population of T cells is invaluable for finding the source of autoimmune diseases and studying immune responses to cancer. But it's a laborious and time-consuming process, as researchers are faced with the prospect of sifting through millions upon millions of possible matches between TCRs and their prospective antigen epitopes—the part of the antigenic molecule to which the receptor binds. Now, as they report in this issue of *PLoS Biology*, Frances Crawford and colleagues have developed a novel method for rapidly identifying TCR mimotopes—peptide sequences similar or identical to epitopes that also elicit the immune response—which can be used to determine the antigen of a given T cell population.

Working backwards, the team started off with two different T cell clones that had been previously selected for with a known antigen—a peptide called p3K. One clone was derived from mice genetically engineered to have broadly reactive T cells; the other, a conventional clone, was much more sensitive to the precise molecular structure of p3K.

Crawford and colleagues then created a “peptide library” comprising more than 30,000 baculoviruses (viruses that selectively target insect cells), each one carrying a slightly different version of the p3K gene, varied in regions of the peptide known to be important for TCR binding. These p3K genes were embedded within a major histocompatibility complex (MHC) gene—a type of cell surface protein that holds displayed antigens and is also important for proper TCR recognition. The team then unleashed their virus library onto insect cells that, once infected, began to produce the specific peptide–MHC complexes encoded on the viral DNA. The insect cells then shuttled these proteins to their surfaces, resulting in a vast array of cells that each displayed a unique variant of the p3K–MHC complex. This “display library” was then incubated with fluorescently labeled TCRs from the two different clones. By observing and isolating the insect cells that lit up, the researchers could see which of the thousands of cells displaying peptide–MHC possessed a mimotope capable of binding a TCR. Because the genetic information about the displayed complex was still stored within the virus-infected cell, the researchers could determine the full peptide sequence responsible for the identified mimotopes.

Confirming the effectiveness of their method, the results of the fluorescence experiments echoed the authors' original characterizations about the two populations of T cells. The broadly reactive TCR bound to several different uniquely displayed complexes; it had 20 mimotopes. The conventional TCR, however, bound only to one peptide–MHC complex, an almost perfect match to the original p3K peptide. Though this study was based on a known antigen and epitope (which allowed verification of the method), the baculovirus display library technique described here could easily be used on T cell populations with unknown antigens. With such a tool, researchers could, for example, identify the antigens connected with tumor-fighting T cells and, through inoculation, possibly induce the production of similar T cells in cancer patients who lack them.

